# Bee Venom-Induced Immunologic Myocardial Infarction: A Rare Case of Kounis Syndrome

**DOI:** 10.1016/j.jaccas.2026.110181

**Published:** 2026-09-02

**Authors:** Gonzalo J. Martinez-Ruiz, Nickolle Cruz-Figueroa, Gerardo E. Martinez-Arroyo, Bryan O. Sepulveda-Bahamundi, Jean C. Rivera-Rivera, Abel E. Rivera-Roman, Coral Del Mar Garcia-Velazquez, Bryan Rodriguez-Melendez, Jorge J. Ballester-Maldonado, Jonathan Cordero-Jimenez

**Affiliations:** a UCC School of Medicine Internal Medicine Residency Program, Hospital Pavia Arecibo, Arecibo, Puerto Rico, USA; b Department of Cardiology, Hospital Pavia Arecibo, Arecibo, Puerto Rico, USA; c Department of Interventional Cardiology, Hospital Pavia Arecibo, Arecibo, Puerto Rico, USA

**Keywords:** acute coronary syndrome, chest pain, coronary angiography, myocardial ischemia

## Abstract

**Background:**

Kounis syndrome is a rare cause of acute coronary syndrome (ACS) triggered by hypersensitivity reactions. Bee venom is a recognized precipitant because of its potent allergenic proteins.

**Case Summary:**

A 50-year-old woman developed urticaria, angioedema, and severe chest pain minutes after a bee sting. Troponins rose exponentially, electrocardiogram demonstrated nonspecific ST segment changes, echocardiography revealed anterior wall hypokinesis, and angiography demonstrated nonobstructive coronary arteries. She was treated with anaphylaxis-directed therapy and vasospasm management, with full recovery.

**Discussion:**

This case highlights the diagnostic and therapeutic challenges of Kounis syndrome, a frequently underrecognized cause of ACS. Current American College of Cardiology/American Heart Association and European Society of Cardiology guidelines do not provide disease-specific recommendations, requiring clinicians to balance anaphylaxis treatment with established ACS therapies. Early recognition is essential to avoid misdiagnosis and guide appropriate management.

**Take-Home Messages:**

Suspect Kounis syndrome when chest pain coincides with allergic reactions. Early recognition enables appropriate treatment and helps prevent misdiagnosis.

## Visual Summary

**Figure:**
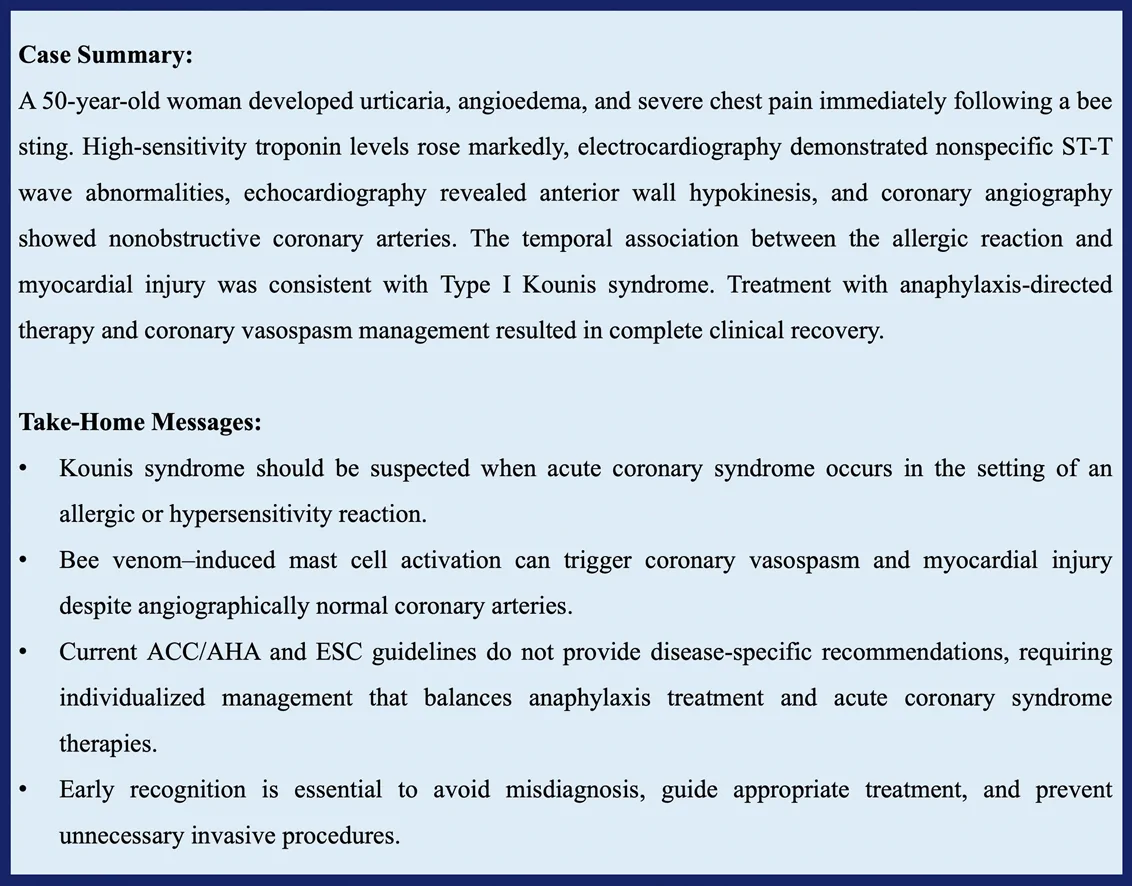

## History of Presentation

A 50-year-old woman with a history of hypertension and hypothyroidism presented to the emergency department with acute chest pain after sustaining a bee sting to the posterior neck while washing her car. Within minutes, she developed diffuse urticaria, periorbital swelling, facial edema, dizziness, vomiting, and severe substernal chest pain radiating to the left arm.

On arrival, she was anxious and in acute distress, but vital signs were stable. Physical examination revealed diffuse urticaria, periorbital edema, and mild jugular venous distension. Lung auscultation demonstrated diffuse wheezing, and cardiac exam showed a regular rhythm without murmurs.

## Past Medical History

• Well-controlled hypertension
• Hypothyroidism.

## Differential Diagnosis

• Acute coronary syndrome (ACS): supported by chest pain.
• Stress cardiomyopathy (Takotsubo): possible in the setting of catecholamine surge.
• Pulmonary embolism: considered given chest pain and tachypnea, but less likely with concurrent rash and allergen exposure.
• Acute viral or hypersensitivity myocarditis: supported by chest pain.
• Coronary vasospasm: supported by chest pain.
• Kounis syndrome: strongly suggested by the immediate temporal association between sting, allergic symptoms, and chest pain.

## Investigations

• Electrocardiography: sinus rhythm with left bundle branch block and secondary repolarization abnormalities, including asymmetric T-wave changes in precordial leads V_1_ and V_2_, together with nonspecific ST-segment abnormalities (Figure 1).

**Figure 1:**
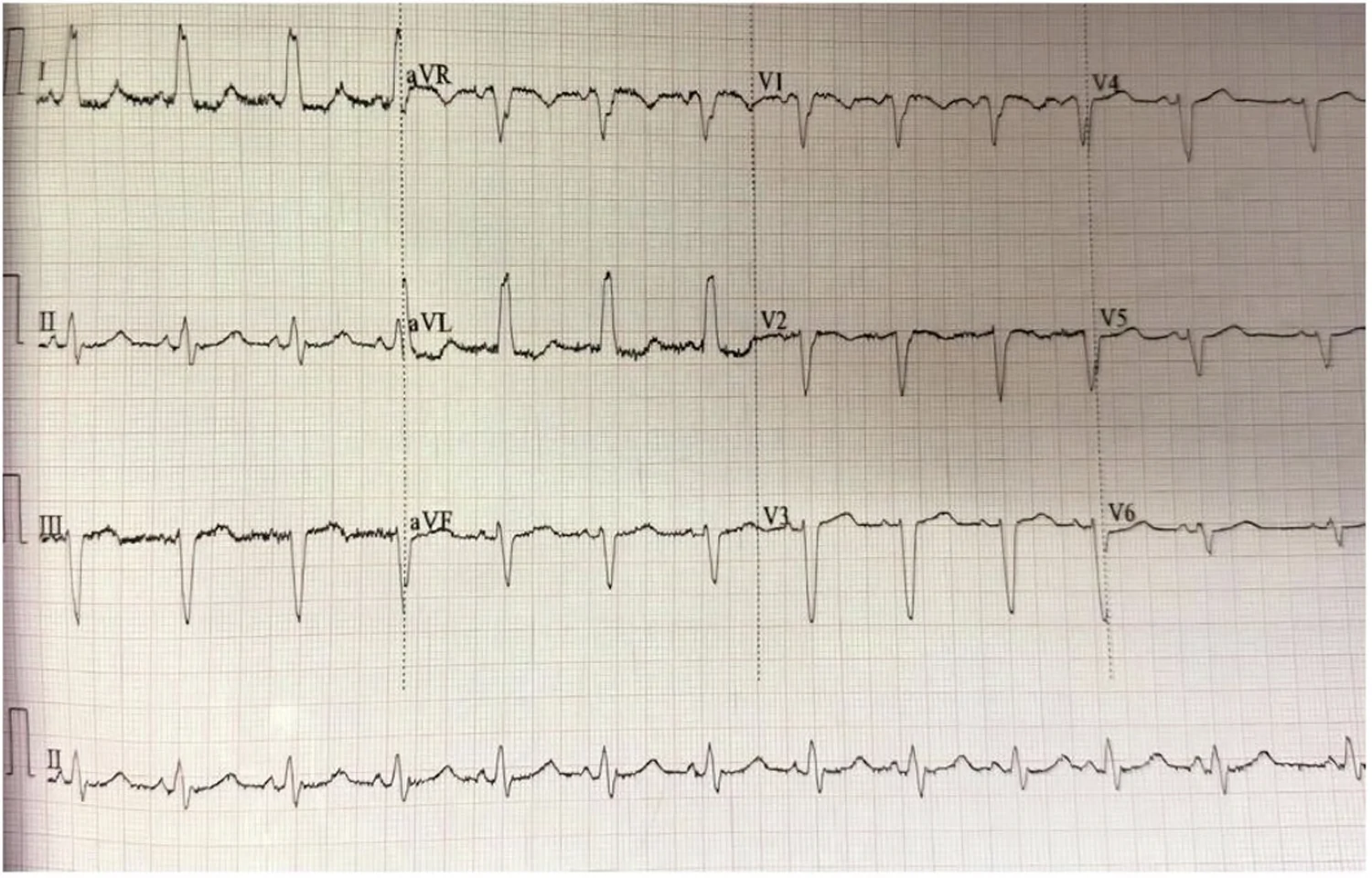
12-Lead Electrocardiogram Sinus rhythm with left bundle branch block and secondary repolarization abnormalities, including asymmetric T-wave changes in precordial leads V_1_ and V_2_, together with nonspecific ST segment abnormalities.
• Laboratory: Leukocytosis 17.5 × 10^9^/L with mild eosinophilia (absolute count 0.70 × 10^3^/μL); hemoglobin, electrolytes, and renal function within normal limits. Lipid panel unremarkable.
• High-sensitivity troponin rose from 201.6 ng/L to 951.1 ng/L, peaked at 1,836.8 ng/L and subsequently declined to 1,308.9 ng/L. Brain natriuretic peptide was elevated at 809 pg/mL (Figure 2).

**Figure 2:**
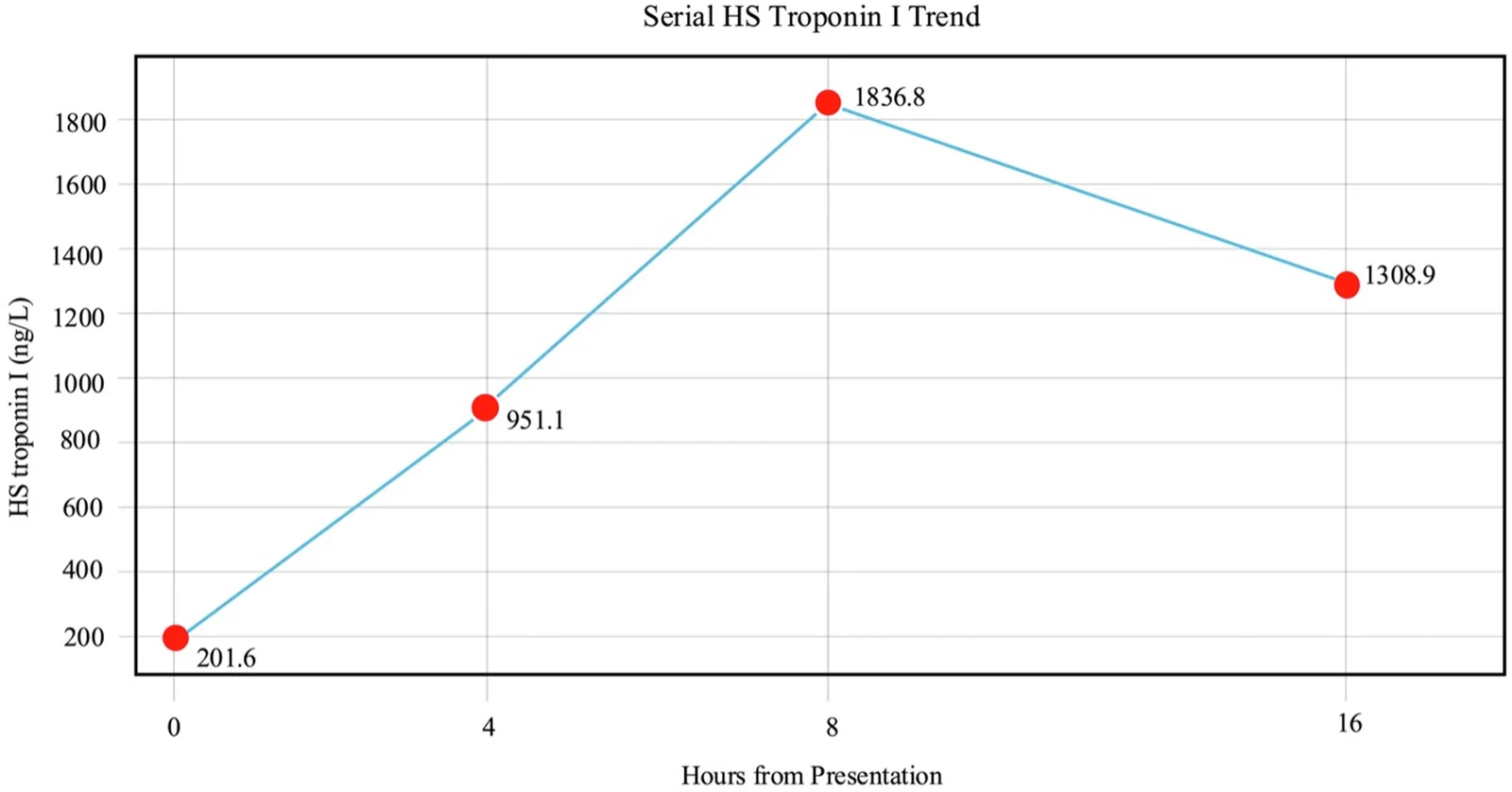
Serial High-Sensitivity Troponin I Levels Graphs Serial high-sensitivity troponin I levels. Values at 0 hours (201.6 ng/L), 4 hours (951.1 ng/L), 8 hours (1,836.8 ng/L), and 16 hours (1,308.9 ng/L) demonstrate a dynamic rise consistent with acute myocardial injury. The linear representation of time vs biomarker levels supports the diagnosis of acute coronary syndrome in the absence of obstructive coronary artery disease, suggesting Type I Kounis syndrome. HS = high-sensitivity.
• Chest radiography: Bilateral perihilar opacities and small left pleural effusion (Figure 3).

**Figure 3:**
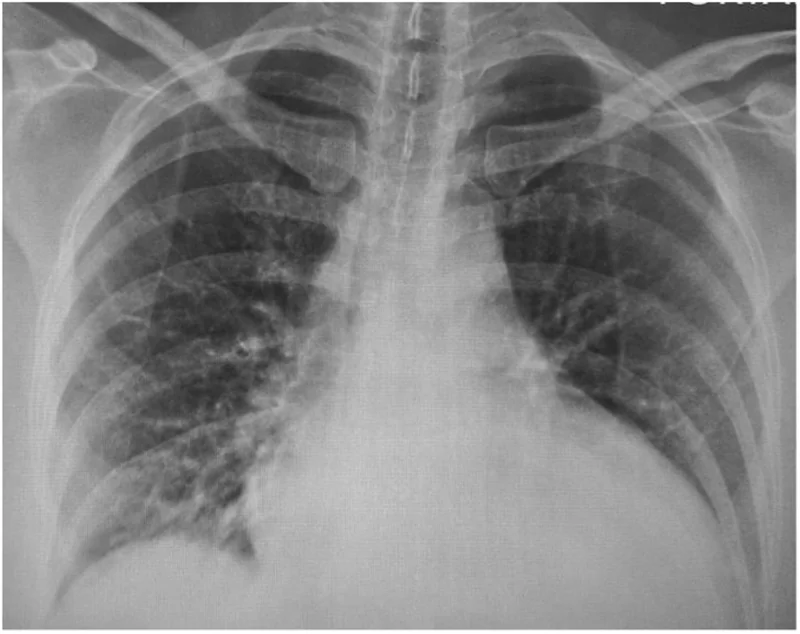
Portable Chest Radiograph Portable chest radiograph demonstrating bilateral perihilar interstitial and alveolar opacities. These findings are consistent with pulmonary edema.
• Transthoracic echocardiography: Mildly reduced left ventricular systolic function (40%-45%), anterior wall hypokinesis, and left atrial enlargement. (Figure 4, Video 1).

**Figure 4:**
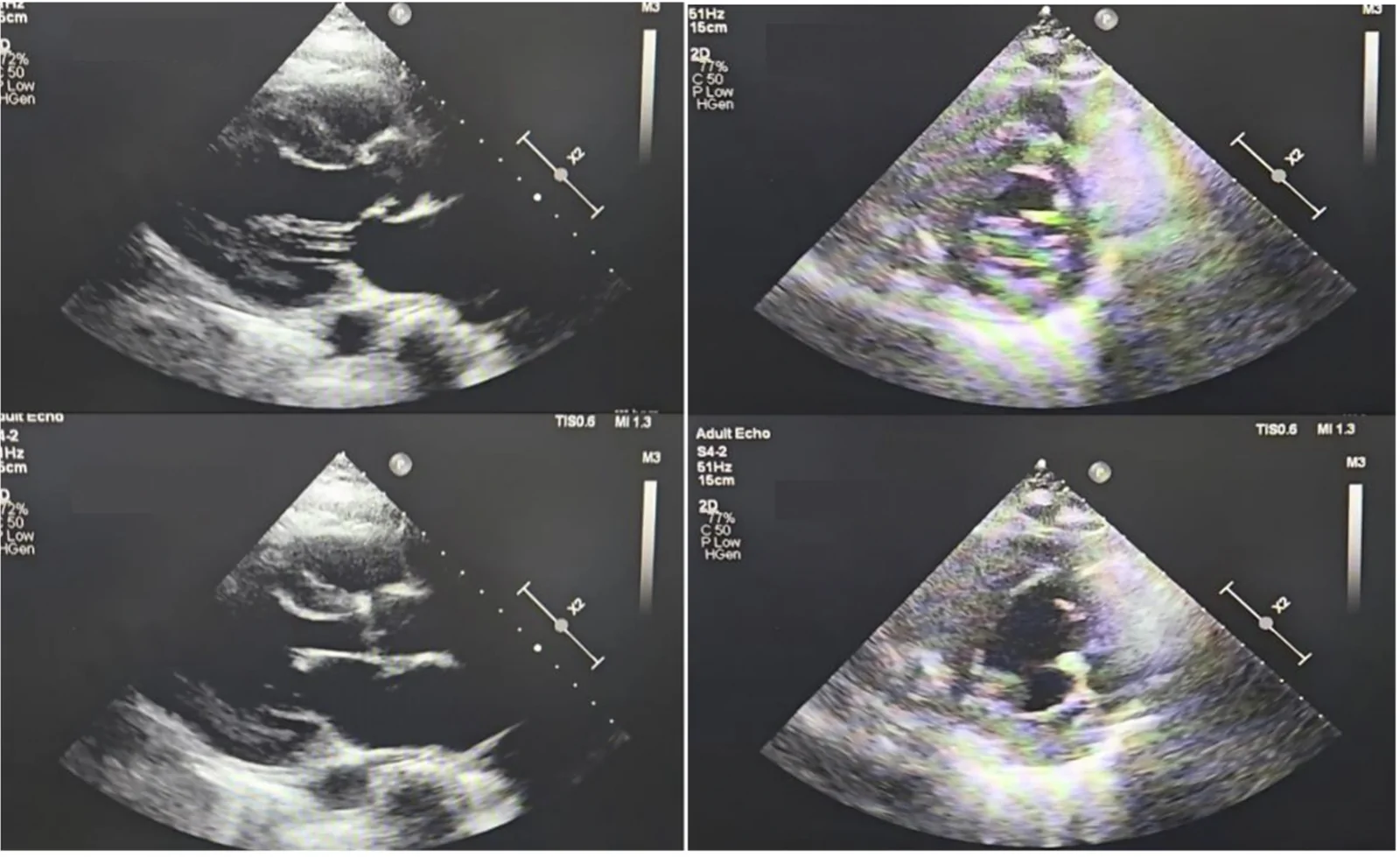
Transthoracic Echocardiogram Transthoracic echocardiogram, parasternal long-axis view (left), and short axis (right) showing mildly reduced left ventricular systolic function with hypokinesis of the anterior wall. The left atrium appears enlarged, and there is no significant pericardial effusion. 2D = 2-dimensional.
• Coronary angiography: Patent left main, left anterior descending artery, and right coronary artery without obstructive lesions. Mild pulmonary hypertension and elevated left ventricular end diastolic pressure were observed (Figures 5 and 6, Video 2).

**Figure 5:**
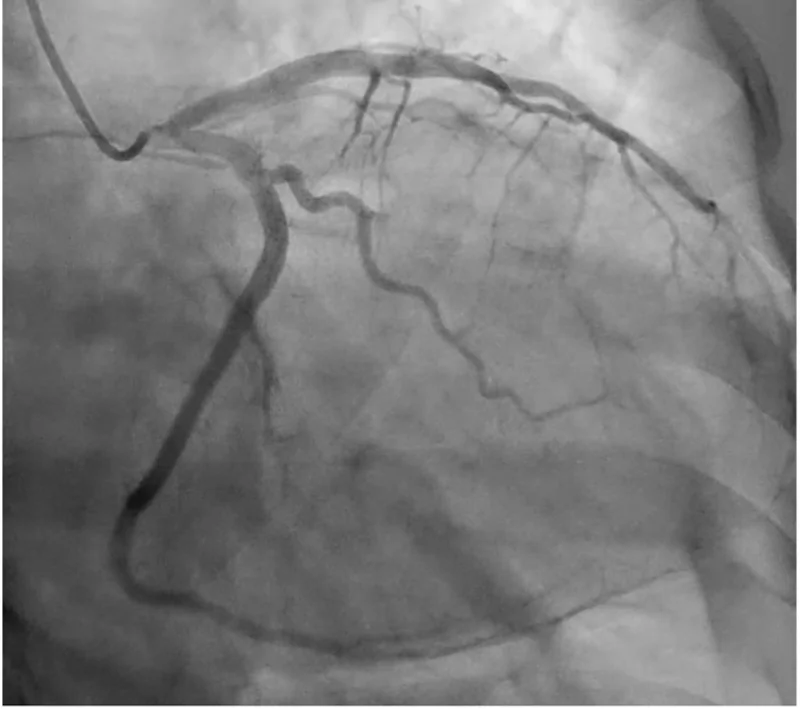
Coronary Angiography Coronary angiography of the left coronary system showing patent left main and left anterior descending artery without obstructive lesion.

**Figure 6:**
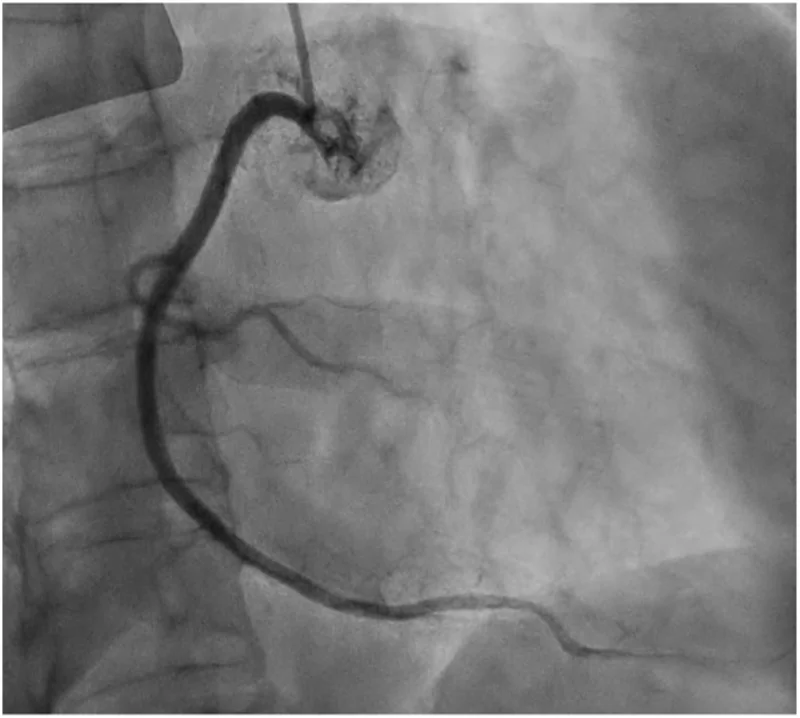
Coronary Angiography Coronary angiography of the right coronary artery demonstrating a normal caliber vessel without fixed stenosis or thrombus. No obstructive coronary artery disease was identified.

## Management

Initial therapy targeted both anaphylaxis and possible ACS. The patient received intramuscular epinephrine (0.3 mg), intravenous methylprednisolone (125 mg), intravenous diphenhydramine (50 mg), aspirin (325 mg loading dose, then 81 mg daily), clopidogrel (300 mg loading dose, then 75 mg daily), therapeutic enoxaparin (1 mg/kg every 12 hours), and intravenous nitroglycerin infusion.

After coronary angiography confirmed normal coronaries, anticoagulation was discontinued. Management was refocused on vasospasm control with nitrates and calcium channel blockers. β-blockers were avoided to prevent worsening vasospasm and interference with epinephrine. Morphine was also avoided because of the risk of histamine release. The patient remained under joint cardiology and allergy/immunology supervision for the remainder of her admission.

## Outcome and Follow-Up

Chest pain resolved within 48 hours. She was discharged on a calcium channel blocker, nitrate, and angiotensin-converting enzyme inhibitor. An epinephrine autoinjector was also prescribed. Allergy follow-up was arranged for venom immunotherapy evaluation. At 3-month follow-up, she remained asymptomatic, and electrocardiogram (ECG) demonstrated resolution of abnormal ST-segment findings and repeated echocardiogram showed improvement in ventricular function.

## Discussion

### Background

Kounis syndrome, first described in 1991, is an ACS triggered by allergic or hypersensitivity reactions. Three variants have been described: Type I, characterized by coronary vasospasm in angiographically normal arteries; Type II, involving plaque erosion or rupture in pre-existing atherosclerotic disease; and Type III, associated with coronary stent thrombosis.^1^^,^^2^ Our patient most likely represents Type I Kounis syndrome, in which mast cell–mediated release of inflammatory mediators induced coronary vasospasm and myocardial injury despite the absence of obstructive coronary artery disease.^3^

### Pathophysiology

Bee venom contains allergenic proteins, including phospholipase A2, melittin, and hyaluronidase, which can trigger mast cell and basophil activation with release of histamine, leukotrienes, prostaglandins, and platelet-activating factor.^4^^,^^5^ These mediators promote coronary vasoconstriction, endothelial dysfunction, and platelet activation, explaining myocardial ischemia, troponin elevation, and transient regional wall-motion abnormalities in the absence of fixed coronary stenosis.^4^^,^^6^, ^7^

### Differential Diagnosis

Although the clinical presentation strongly suggested Type I Kounis syndrome, the diagnosis remains presumptive, and other causes of myocardial infarction with nonobstructive coronary arteries (MINOCA) should be considered.^8^ Acute viral myocarditis, hypersensitivity myocarditis, Takotsubo syndrome, coronary thromboembolism, and spontaneous coronary vasospasm unrelated to allergic reactions were considered in the differential diagnosis.^8^^,^^9^ Hypersensitivity myocarditis deserves consideration because it may also occur following allergic reactions and present with chest pain, elevated cardiac biomarkers, and transient left ventricular dysfunction. However, the immediate onset of chest pain within minutes of the bee sting, concomitant urticaria and angioedema, objective evidence of myocardial injury, transient regional wall-motion abnormalities, and angiographically normal coronary arteries favored presumed Type I Kounis syndrome.^1^^,^^3^ Takotsubo syndrome was considered less likely because echocardiography demonstrated focal anterior wall hypokinesis rather than the characteristic apical or midventricular ballooning pattern.^8^ Coronary vasospasm was not directly demonstrated angiographically but was inferred from the clinical presentation in the absence of obstructive coronary artery disease.

### Management

Current American College of Cardiology/American Heart Association and European Society of Cardiology guidelines for ACSs and chest pain do not provide disease-specific recommendations for Kounis syndrome. Consequently, management requires balancing the priorities of anaphylaxis treatment with established ACS therapies.

• Epinephrine: Remains the cornerstone of anaphylaxis management but should be administered with caution because it may exacerbate coronary vasospasm and myocardial ischemia.
• ACS therapy: Anticoagulation and dual antiplatelet therapy are reasonable until obstructive disease is excluded.
• Anti-ischemic therapy: Nitrates and calcium channel blockers are preferred for the treatment of coronary vasospasm, whereas β-blockers and morphine should generally be avoided because they may worsen vasospasm or amplify allergic mediator release.^5^

The absence of formal guideline recommendations highlights the importance of maintaining a high index of suspicion and adopting a multidisciplinary approach to diagnosis and management.

### Preventive measures

Preventive strategies include allergen avoidance, prescription of epinephrine autoinjectors, and referral for allergy/immunology evaluation, including consideration of venom immunotherapy. Long-term cardiology follow-up is also important to document recovery of ventricular function and guide ongoing cardiovascular risk management.

### Study Limitations

Several limitations should be acknowledged. Cardiac magnetic resonance imaging, an important diagnostic modality in the evaluation of MINOCA, was not available at our institution and therefore was not performed. Consequently, microvascular ischemia, myocarditis, and other inflammatory causes of MINOCA could not be definitively excluded, limiting diagnostic certainty.^9^ Serum tryptase and total immunoglobulin E levels were not obtained during the acute presentation and therefore additional evidence of mast-cell activation could not be assessed. Previous ECGs were unavailable, precluding determination of whether the left bundle branch block was chronic or new. Furthermore, because left bundle branch block is associated with secondary repolarization abnormalities, the asymmetric T-wave changes observed on the admission ECG should be interpreted cautiously and were not considered diagnostic of myocardial ischemia in isolation.

### Clinical Importance

Although underrecognized, registry data estimate that Kounis syndrome occurs in approximately 1.1% of hospitalizations for allergic, hypersensitivity, or anaphylactic reactions.^4^ Its true incidence may be higher because of underdiagnosis and overlap with other causes of ACS. Failure to recognize Kounis syndrome may lead to unnecessary interventions, prolonged antithrombotic therapy, or inappropriate medication selection; conversely, delayed treatment of anaphylaxis may be life-threatening.

### Educational Value

This case highlights the need for awareness among emergency physicians, hospitalists, and cardiologists. Recognizing the overlap between allergic and cardiovascular presentations is essential to avoid misdiagnosis and guide appropriate therapy. Incorporating Kounis syndrome into the differential diagnosis of ACS, particularly after allergic exposures, may improve diagnostic accuracy and prevent mismanagement.

## Conclusions

This case demonstrates that ACSs may arise from immunologic mechanisms, not solely from atherosclerosis, highlighting the complex interplay between the immune and cardiovascular systems. Recognition of this entity is essential because its presentation may closely mimic conventional myocardial infarction, yet its underlying pathophysiology and management differ substantially. Increased awareness of Kounis syndrome can facilitate timely diagnosis, appropriate treatment, and improved patient outcomes while avoiding unnecessary invasive procedures and prolonged antithrombotic therapy.

**Visual Summary:**
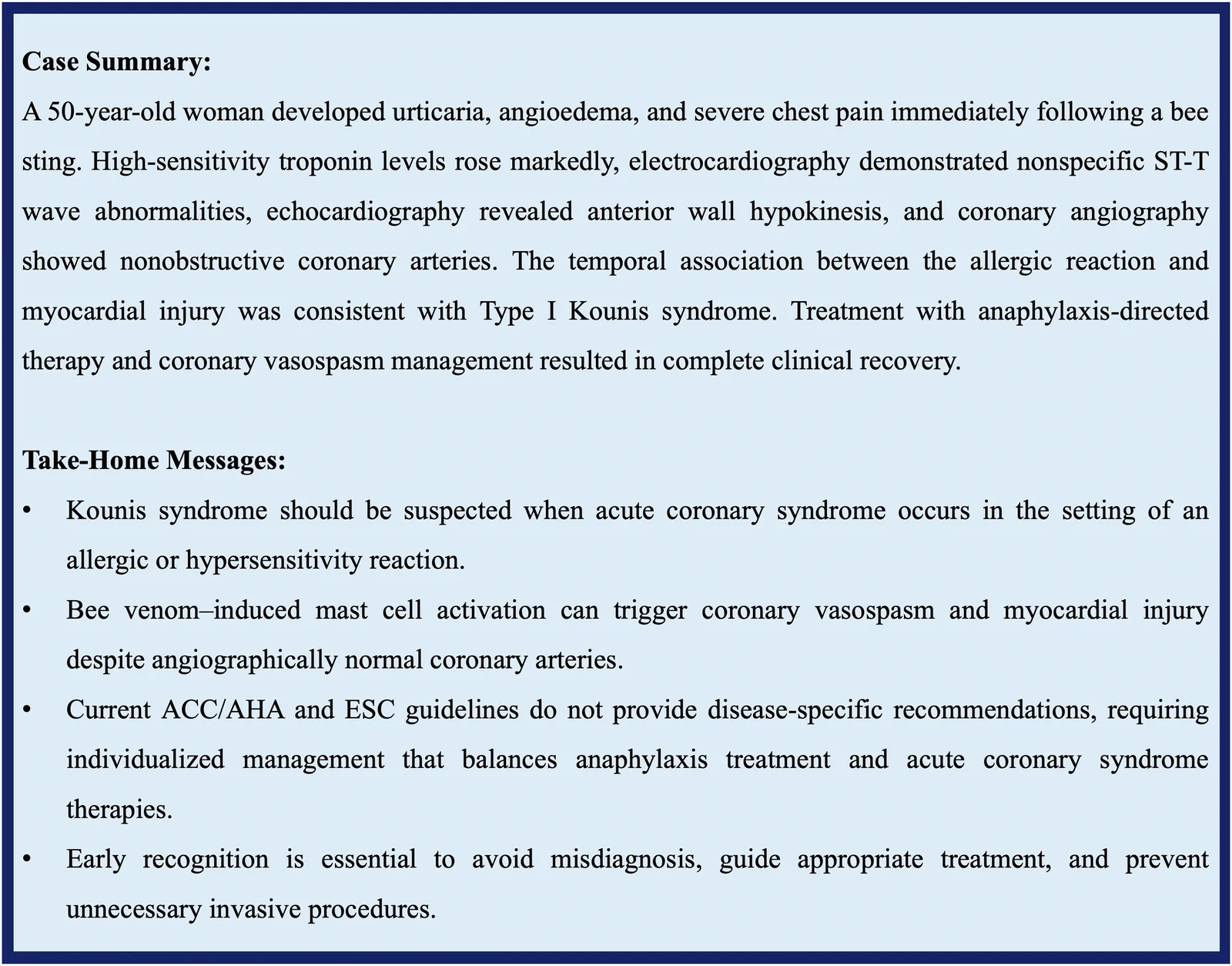
Visual Overview of the Patient’s Clinical Presentation, Diagnostic Evaluation, Management, and Clinical Outcome in Type I Kounis Syndrome Following a Bee Sting The summary illustrates the temporal relationship between the allergic reaction and the development of acute coronary syndrome, the diagnostic workup leading to the diagnosis, and the therapeutic approach. It highlights the importance of including Kounis syndrome in the differential diagnosis of acute coronary syndrome when cardiac symptoms occur in temporal association with an allergic reaction and emphasizes an individualized management strategy addressing both the hypersensitivity reaction and myocardial ischemia. ACC = American College of Cardiology; AHA = American Heart Association; ESC = European Society of Cardiology.

## Funding Support and Author Disclosures

The authors have reported that they have no relationships relevant to the contents of this paper to disclose.

**Take-Home Messages**

• Not all acute coronary syndromes are caused by atherosclerotic plaque rupture; allergic and inflammatory mechanisms should be considered when ischemic symptoms occur in the setting of anaphylaxis or hypersensitivity reactions.
• Early recognition of Kounis syndrome is critical to guide appropriate treatment and avoid interventions that may exacerbate coronary vasospasm or myocardial ischemia.

## Appendix

Video 1 Transthoracic Echocardiogram Video Transthoracic echocardiography demonstrating mildly reduced left ventricular systolic function with hypokinesis of the anterior wall. The left atrium is mildly enlarged, and no significant pericardial effusion is identified.

Video 2 Coronary Angiography Video Coronary angiography of the right and left coronary systems demonstrating patent left main, left anterior descending, and right coronary arteries without obstructive coronary artery disease, angiographic evidence of plaque rupture, or intracoronary thrombus. Mild pulmonary hypertension and an elevated left ventricular end-diastolic pressure were also noted during invasive hemodynamic assessment.
